# *DENND5B* Regulates Intestinal Triglyceride Absorption and Body Mass

**DOI:** 10.1038/s41598-019-40296-0

**Published:** 2019-03-05

**Authors:** Scott M. Gordon, Edward B. Neufeld, Zhihong Yang, Milton Pryor, Lita A. Freeman, Xiao Fan, Iftikhar J. Kullo, Leslie G. Biesecker, Alan T. Remaley

**Affiliations:** 1 Translational Vascular Medicine Branch, National Heart, Lung, and Blood Institute, NIH, Bethesda, Maryland 20892 USA; 2 Saha Cardiovascular Research Center and Department of Physiology, University of Kentucky College of Medicine, Lexington, KY 40536 USA; 3 Department of Cardiovascular Diseases, Mayo Clinic, Rochester, Minnesota 55905 USA; 4 Medical Genomics and Metabolic Genetics Branch, National Human Genome Research Institute, NIH, Bethesda, Maryland 20892 USA

## Abstract

Regulation of lipid absorption by enterocytes can influence metabolic status in humans and contribute to obesity and related complications. The intracellular steps of chylomicron biogenesis and transport from the Endoplasmic Reticulum (ER) to the Golgi complex have been described, but the mechanisms for post-Golgi transport and secretion of chylomicrons have not been identified. Using a newly generated *Dennd5b*^−/−^ mouse, we demonstrate an essential role for this gene in Golgi to plasma membrane transport of chylomicron secretory vesicles. In mice, loss of *Dennd5b* results in resistance to western diet induced obesity, changes in plasma lipids, and reduced aortic atherosclerosis. In humans, two independent exome sequencing studies reveal that a common *DENND5B* variant, p.(R52K), is correlated with body mass index. These studies establish an important role for *DENND5B* in post-Golgi chylomicron secretion and a subsequent influence on body composition and peripheral lipoprotein metabolism.

## Introduction

Obesity is a major risk factor for cardiovascular disease (CVD) and its prevalence is rapidly growing worldwide^1^. Despite effective lowering of plasma lipids with statins, obese patients often show progression of atherosclerotic disease, whereas non-obese individuals typically show regression^2^. It is currently unclear whether this increased risk is a direct consequence of increased body mass or is due to co-morbidities often associated with obesity that are well known risk factors for CVD, such as hypertension, dyslipidemia, and type 2 diabetes^3^. Genetic factors that influence body weight may prove to be effective targets for interventional reduction of body weight and cardiovascular comorbidities.

Current, pharmacological interventions for obesity have largely been aimed at suppressing appetite and blockade of fat absorption^4^. Intracellular trafficking of triacylglyceride (TG) in the formation and secretion of chylomicrons is an area of great interest, because of the potential to reduce absorption of dietary fat. The pathways and molecular machinery involved in fatty acid absorption, re-esterification of TG, chylomicron biogenesis and endoplasmic reticulum (ER) to Golgi transport have been well characterized^5^. The importance of these pathways in human energy metabolism is demonstrated in patients with pathogenic variants in *APOB*, *MTP*, and *SAR1B* (formerly *SARA2*)^6^. *APOB* and *MTP* variants can affect the initial formation of chylomicrons in the enterocyte, and SAR1B is a GTPase required for ER to Golgi movement of prechylomicron transport vesicles. Pathogenic variants in *SAR1B* result in chylomicron retention disease, a fat absorption defect characterized by dietary fat-intolerance and failure to thrive in infancy^7^. Less is known about the downstream chylomicron secretory processes by which mature chylomicrons are packaged by the Golgi, transported to the plasma membrane, and secreted. A more complete understanding of intracellular fat absorption pathways may guide better-targeted therapies with fewer side effects.

During a phage display screen, our lab identified a DENN family member, *DENND5B*, as a possible lipoprotein binding protein. This gene codes for a 145 kDa protein with a predicted transmembrane domain near the C-terminus. The DENN domain-containing family of proteins in humans comprises 18 members, whose physiological functions are largely uncharacterized^8^. The DENN domain itself has guanine nucleotide exchange factor (GEF) activity toward Rab GTPases and the Rab specificity of DENN proteins varies among family members^9^. Based on this function, it has been proposed that DENN proteins are involved in the regulation of intracellular vesicular transport pathways. In mice, Dennd5b is expressed at relatively high levels in liver, small intestine, and brain^10^ (Supplemental Fig. 1A).

In the current study, we generated a *Dennd5b* knockout mouse and found that the mice have decreased absorption of dietary TG due to a post-Golgi defect in chylomicron secretion. Additionally, we tested the hypothesis that *DENND5B* is important in human lipid metabolism using two independent exome sequencing cohorts.

## Methods

### Generation of *Dennd5b*^−/−^ mice

A custom zinc finger nuclease (ZFN) was generated to target an exonic sequence within the first third of the *Dennd5b* gene. The ZFN RNA (20–40 ng/uL) was administered to fertilized B6CBAF1 mouse embryos by pronuclear injection and embryos were implanted into females. Resulting pups were genotyped, using PCR and Sanger sequencing. A *Dennd5b*^−/−^ line with a biallelic 19 bp deletion was established. This mutation predicts a frameshift and early termination due to the introduction of a stop codon. Wildtype mice (WT) on the same background were used as controls for all experiments. Mice were fed standard chow (Envigo 7017) or western diet (WD) with 42% calories from fat (Harlan TD.88137). Western diet studies were initiated in mice beginning at 2 months of age. For all other experiments mice used were between 2–4 months of age and controls were age matched (within one week). All animal protocols and procedures conform to the National Institutes of Health Guide for the Care and Use of Laboratory Animals and were approved by the National Heart Lung and Blood Institute ACUC.

### Blood collection and plasma lipid analyses

For all experiments, mouse blood was collected by retro-orbital bleed, using heparinized capillary tubes and added to tubes containing EDTA. Plasma was obtained by centrifugation at 3,000 rpm for 20 min. at 4 °C. Plasma lipids were measured using colorimetric enzymatic assays (Wako Diagnostics). Size-exclusion chromatography separations of plasma lipoproteins were performed on an Akta Pure instrument equipped with two Superose 6 (GE Healthcare) columns arranged in series. Plasma (100 µL) was run over columns at a flow rate of 0.5 mL/min in Tris buffer (10 mM Tris, 150 mM NaCl, 0.5 mM EDTA, 0.01% sodium azide) and 0.5 mL fractions collected.

### Lipid absorption studies

Mice were fasted overnight (≥16 hours) before lipid absorption experiments. Vegetable oil (10 uL/g body weight) was administered by oral gavage, using a blunt ball-tipped syringe. Plasma was collected as described above, at time points indicated, and triglyceride measured by enzymatic assay.

### VLDL secretion assay

Mice were fasted overnight (16 hours) and baseline blood collected prior to retroorbital injection (6 µL/g body weight) of Tyloxapol in saline (5% wt:vol). Blood was collected from mice by retroorbital bleed from eye opposite to injection site at 1, 2, and 4 hours post-injection. Plasma was analyzed for triglyceride content by enzymatic assay.

### Hepatic lipid analysis

Liver tissue harvested from PBS perfused mice was snap frozen with liquid nitrogen and stored at −80 °C until lipid extraction. A portion of liver (80–100 mg) from the lateral left lobe was used for lipid extraction. Tissue was added to 1 mL 0.9% NaCl, minced and lipids collected by chloroform methanol extraction. Chloroform layer containing lipid was dried under nitrogen gas. Dry lipid was solubilized in hexane isopropanol (3:2, v:v) and a small volume used for measurement of cholesterol, triglyceride, and free fatty acids by enzymatic assay. All measurements were adjusted to the starting liver tissue mass.

### Fecal lipid analysis

Feces were collected from mice over a 3-day period and dried before measuring total mass. About 250 mg of dry feces was pulverized, using a mortar and pestle, and total lipids were isolated by chloroform methanol (2:1, v:v) extraction. Chloroform layer containing lipid was dried under nitrogen gas and weighed to determine total lipid mass. Dry lipid was solubilized in hexane isopropanol (3:2, v:v) and a small volume used for measurement of cholesterol, triglyceride, and free fatty acids by enzymatic assay (All from Wako Diagnostics). All measurements were adjusted to the starting fecal mass.

### Electron microscopy of small intestine

Mice were sacrificed by cervical dislocation and a 1 mm section of duodenal small intestine tissue was quickly harvested beginning 1 mm distal to the pyloric sphincter of the stomach. Tissue was immediately placed in Karnovsky’s fixative and chopped into cubes 1 mm^3^ or less, post-fixed in osmium tetroxide, dehydrated and embedded in Epon for sectioning. Sections on uncoated grids were imaged on a JEOL JEM 1200EXII transmission electron microscope.

### Aortic atherosclerosis lesion area quantification

Mouse aortas, from base of aortic arch to the femoral branch, were harvested and fixed in fresh 4% paraformaldehyde for 5 minutes before staining with Sudan IV (0.5%, wt:vol) for 15 minutes. After staining, aortas were destained for 15 minutes with 80% ethanol and stored in water. Scissors were used to open the aortas for *en face* analyses. Color images were taken along the full length of the aorta and imaging software was used to quantify stained lesions. Atherosclerotic lesion area was calculated as (stain positive area/total aortic surface area)*100.

### *DENND5B* genotype association studies in Humans

The ClinSeq^®^ exome sequencing cohort^11^ comprising 621 participants with reliable sequence data for the *DENND5B* Arginine 52 codon (variant rs4930979), were used for analysis. Body mass and blood lipid parameters were evaluated across homozygous reference, heterozygous, and homozygous variant genotypes for the c.155G > A; p.(R52K) variant by ANOVA with Tukey correction for multiple comparisons.

The Mayo Vascular Disease Biorepository (VDB) was used for replication analyses^12^. Genotyping of 9,274 VDB participants was performed, using genome-wide SNP arrays on Illumina platforms. Genotype data were imputed using Michigan Imputation Server^13^ based on a human reference consortium (HRC, r1.1) panel^14^. Genotype association tests for lipid levels and BMI were based on linear regression analyses assuming an additive effect with adjustment for age, sex and the first two principal components.

The datasets generated during and/or analyzed during the current study are available from the corresponding author on reasonable request.

## Results

### *Dennd5b*^−/−^ mice have low plasma HDL

**Figure 1:**
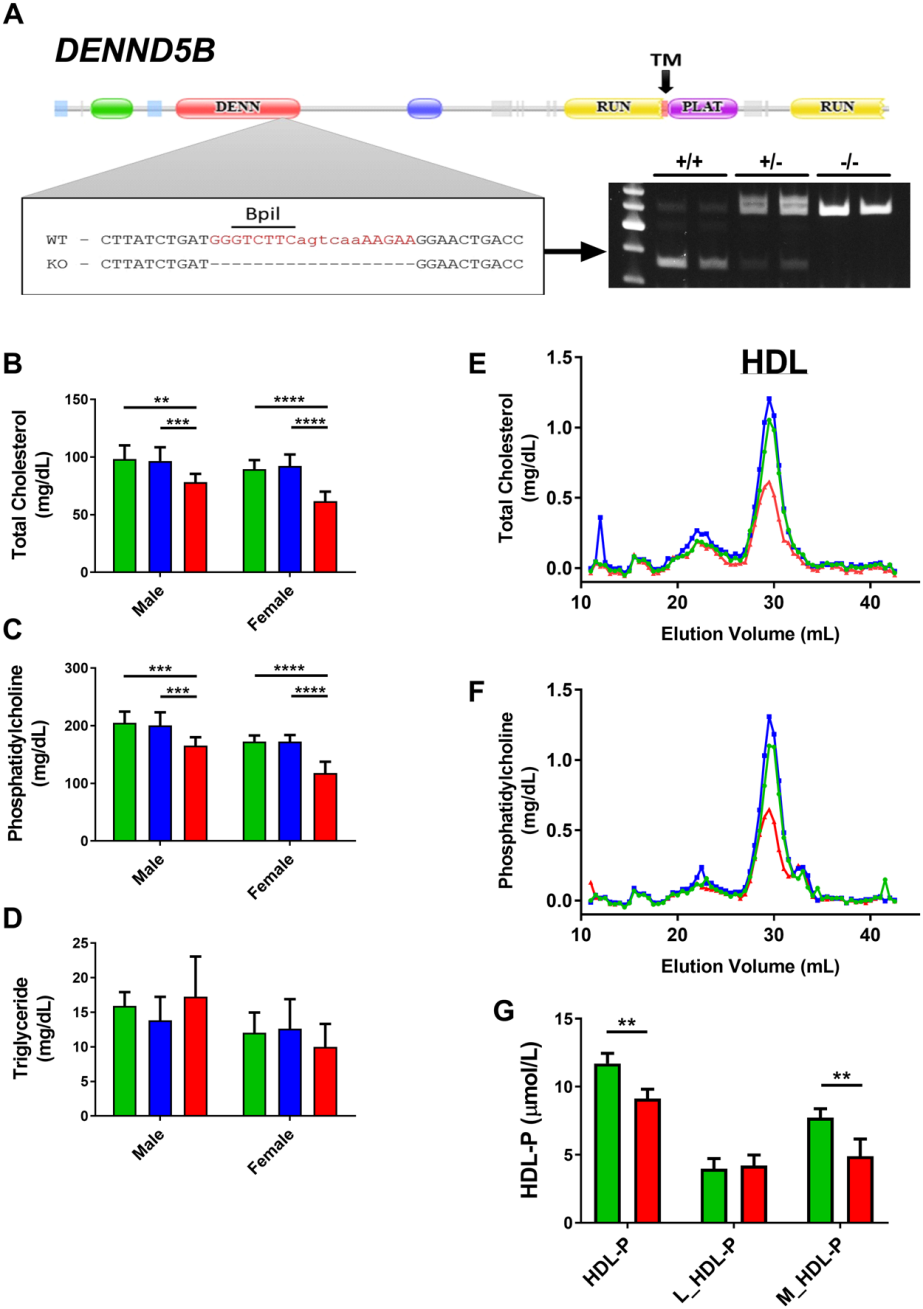
Knockout of *Dennd5b* in mice results in reduced plasma high density lipoprotein lipids and particle number. (**A**) Genetic knockout of *Dennd5b* in mice using zinc-finger nuclease resulted in a 19 bp deletion. Genotyping strategy utilizes PCR amplification of a DNA fragment containing the cut site and exploits loss of Bpil restriction site in mutant mice. (**B–D**) Total cholesterol, phospholipid, and triglyceride in plasma of wildtype (green), heterozygous (blue), and homozygous (red) mice by gender. n = 6–12/group on chow diet. (**E,F**) Size-exclusion chromatography analyses of plasma lipoproteins in wildtype, heterozyous, and homozygous female mice. Each trace represents a pooled sample from n = 3/group on chow diet. (**G**) High density lipoprotein particle number was measured by nuclear magnetic resonance on a Vantera Lipoprotein Analyzer. n = 3 pools of 5 female mice/group on chow diet. Statistical analyses were performed using 2-way ANOVA with Tukey *post hoc* tests (**p < 0.01, ***p < 0.001, ****p < 0.0001). All values are mean ± standard deviation.

A *Dennd5b*^−/−^ mouse line was generated, using a zinc finger nuclease. This approach resulted in a 19 bp deletion (Fig. 1A), predicting a frameshift mutation and early termination signal. Mice were fertile with outwardly normal morphology. Measurement of plasma lipids in *Dennd5b*^−/−^ mice revealed a significant reduction in plasma total cholesterol (TC) and phospholipids (PL) but not triglyceride (TG) (Fig. 1B–D). Reduced lipids were only present in homozygous mice, not in heterozygous, and the effect was greater in females than in males (−30% vs. −20%). Because female *Dennd5b*^−/−^ mice demonstrated a more prominent plasma lipid phenotype, female mice were used for subsequent experiments. Size-exclusion chromatography of mouse plasma demonstrated that the reduction in lipids was attributed entirely to a reduction of HDL-sized lipoproteins (Fig. 1E,F). Nuclear magnetic resonance analyses of lipoprotein particle concentrations indicated a 22% reduction in total HDL particle number (HDL-P), which was entirely due to a lower concentration of medium sized HDL particles (Fig. 1G). Large HDL particle numbers were not affected.

### Impaired intestinal triglyceride absorption in *Dennd5b*^−/−^ mice

**Figure 2:**
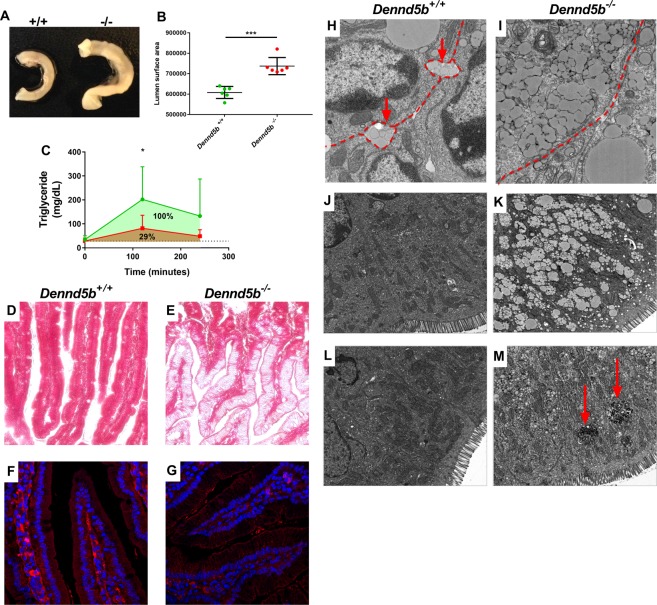
*Dennd5b*^−/−^ mice have enlarged small intestine and reduced triglyceride absorption due to impaired chylomicron secretion by enterocytes. (**A**) Picture of duodenal segments from overnight fasted wildtype and *Dennd5b*^−/−^ mice. (**B**) A 25 mm segment of duodenal small intestine, beginning 20 mm distal to pyloric sphincter, was cut longitudinally and laid flat for imaging and surface area calculation. Lumenal surface area was calculated as length × width of the segment in image pixels. n = 6/group. ***p < 0.0001 by unpaired t-test. (**C**) Mice were given an oral gavage of vegetable oil (10 uL/g of body weight) and appearance of triglyceride in the plasma was measured at baseline, 2, and 4 hours post-gavage. n = 10/group. *p = 0.01 for wildtype vs knockout using 2-way ANOVA with Sidak post hoc test. (**D,E**) Masson stained duodenal sections from wildtype and *Dennd5b*^−/−^ mice 2 hours after oral oil-gavage. (**F,G**) Immunofluorescence stained duodenal sections 2 hours after oral oil-gavage. DAPI nuclear stain (Blue) and anti-apoB (Red). (**H,I**) Electron micrographs of duodenal tissue at one hour after oral oil-gavage. (**J,K**) Electron micrographs of duodenal tissue at two hours after oral oil-gavage. (**L,M**) Electron micrographs of duodenal tissue after an overnight fasting period. All values are mean ± standard deviation. All mice used in these experiments were female.

Gross inspection of all internal organs of the *Dennd5b*^−/−^ mice was normal, except for the small intestine. *Dennd5b*^−/−^ mice on chow diet had a distended small intestine (Fig. 2A), quantified by a 20% increase in luminal surface area (Fig. 2B). The small intestine had a whitish color consistent with possible fat accumulation, even though the mice had been fasting overnight. To evaluate transit of dietary triglyceride to the plasma, oil-gavage studies were performed in four-month-old fasting mice. These studies showed significantly lower plasma triglyceride (Fig. 2C) and free fatty acids at 2 hours post-gavage in *Dennd5b*^−/−^ mice compared to wildtype controls. Microscopic analyses of duodenal sections showed massive lipid accumulation in *Dennd5b*^−/−^ enterocytes (Fig. 2D,E). Additionally, immunofluorescence confocal microscopy of duodenal sections indicated that secretion of apoB into the lacteal was dramatically lower in *Dennd5b*^−/−^ small intestine (Fig. 2F,G). Taken together, these data suggest a significant impairment of intestinal TG absorption in *Dennd5b*^−/−^ mice.

### Dennd5b is involved in post-Golgi chylomicron secretion by enterocytes

To gain insight into the mechanism of the involvement of Dennd5b in intestinal TG absorption, transmission electron microscopy was used to examine duodenal enterocytes in wildtype (WT) and *Dennd5b*^−/−^ mice. Imaging of tissue at various time points after oral oil gavage allowed for visualization of the sequential steps of chylomicron secretion. At 1 hour post-gavage, fusion events between chylomicron secretory vesicles and the plasma membrane result in secretion of chylomicrons by WT enterocytes (Fig. 2H). In *Dennd5b*^−/−^ mice, these fusion events are rare and very few extracellular chylomicrons are observed (Fig. 2I). At 2 hours, WT enterocytes have cleared the majority of lipid (Fig. 2J); however, there is significant accumulation of fat in *Dennd5b*^−/−^ enterocytes in the form of vesicle-bound chylomicrons and some lipid droplets (Fig. 2K). A more detailed series of electron micrographs of enterocytes taken at various time points after oral oil gavage are presented in Supplemental Fig. 2. These images revealed similar lipid droplet formation, pre-chylomicron formation in the ER lumen, pre-chylomicron transport vesicles (PCTV’s), chylomicron maturation in the Golgi and budding of chylomicron secretory vesicles (CSVs) from the Golgi in wildtype and *Dennd5b*^−/−^ mice. However, in contrast to wildtype mice, *Dennd5b*^−/−^ enterocytes exhibited massive accumulation of CSV’s, which appeared to be unable to fuse with the basolateral plasma membrane. Whereas WT mice secreted chylomicrons into the intercellular space and the lamina propria by 1 hour after gavage, chylomicron secretion by *Dennd5b*^−/−^ enterocytes was rarely observed. Lower magnification electron micrographs allowed for visualization of the extent of lipid accumulation in the enterocyte (Supplemental Fig. 3). Together, these findings support a role for Dennd5b in post-Golgi transport of chylomicron secretory vesicles. Enteric lipid accumulation was even observed in *Dennd5b*^−/−^ mice that did not receive an oil gavage and had undergone an overnight period of fasting (Fig. 2L,M). In *Dennd5b*^−/−^ enterocytes, electron dense structures similar to intracellular digestive vesicles were observed (Fig. 2M, red arrow and Supplementary Fig. 4), which may represent an alternative pathway for the removal of excess intracellular TG by autophagy.

### *Dennd5b*^−/−^ mice are resistant to western diet induced weight gain and changes in plasma lipids

**Figure 3:**
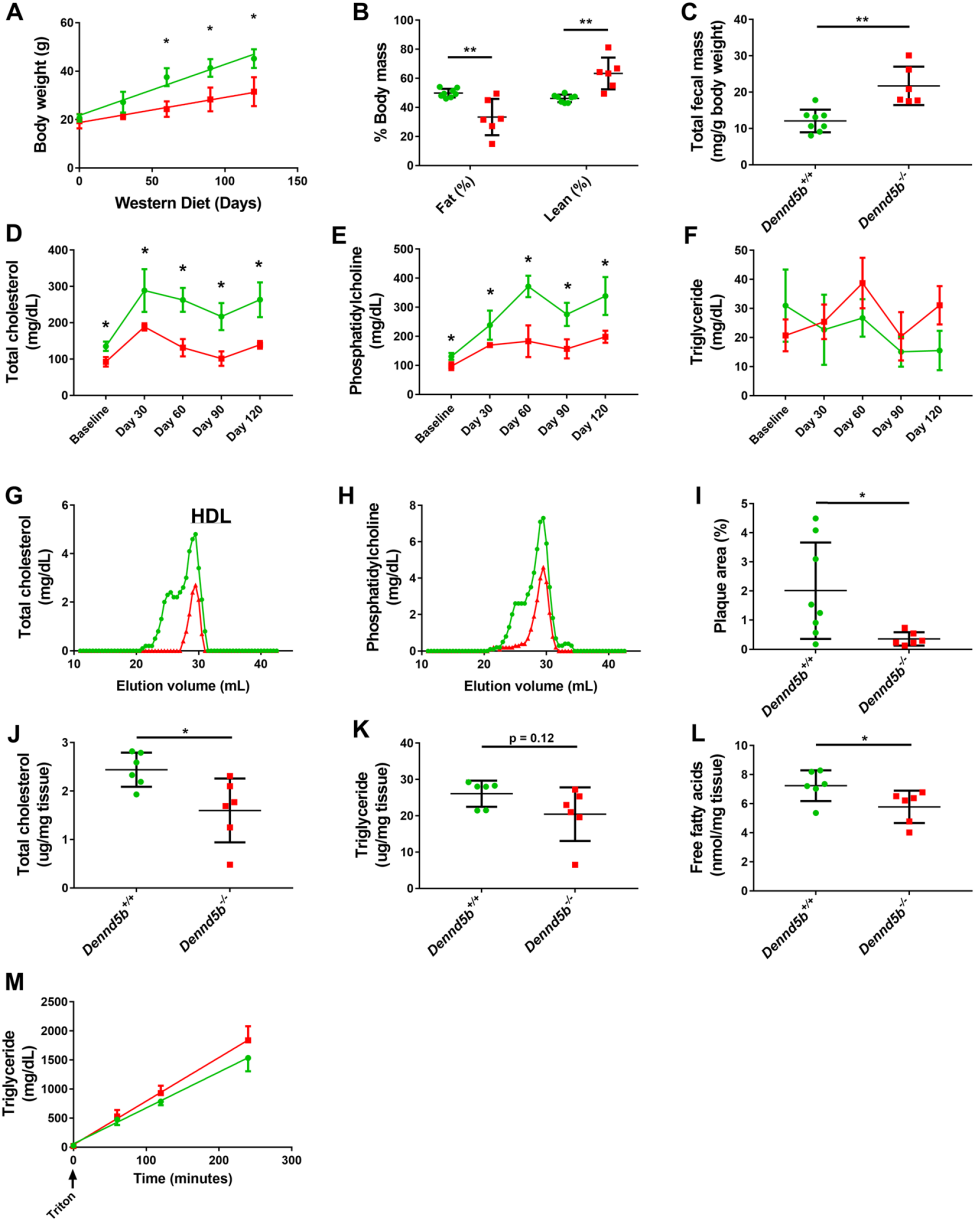
Knockout of *Dennd5b* confers resistance to western diet induced weight gain, plasma and liver lipid increases, and atherosclerotic lesion development. (**A**) Body weights of wildtype and *Dennd5b*^−/−^ mice during 4-months on western diet. n = 6/group. *p < 0.0001 by 2-way ANOVA with Sidak post hoc test. (**B**) NMR body composition analyses of wildtype and *Dennd5b*^−/−^ mice after 4-months on western diet. n = 6/group. **p < 0.01 by 2-way ANOVA with Sidak post hoc test. (**C**) Feces were collected from mice over a 3-day period and dried before measuring total mass. n = 6–8/group. **p < 0.01 by unpaired t-test. (**D**–**F**) Plasma total cholesterol, phospholipid, and triglyceride were measured during 4-months on western diet. *p < 0.01 t-test with multiple comparisons correction by the Holm-Sidak method. (**G**–**H**) Size-exclusion chromatography analyses of plasma lipoproteins in wildtype and homozygous mice after 4-months on western diet. Each trace represents a pooled sample from n = 3/group. (**I**) Quantification of lipid rich atherosclerotic lesions in the aortas of mice after 4-months on western diet. Aortas were harvested, stained with Sudan IV, and mounted for *en face* analyses. Plaque area was calculated as % of total aorta area cover by positive Sudan IV staining. *p < 0.05 by unpaired t-test. (**J**–**L**) Lipids were extracted from liver after 4-months on western diet and total cholesterol, triglyceride, and free fatty acids were measured. n = 6/group. *p < 0.05 by unpaired t-test. (**M**) VLDL production was measured by giving mice a retroorbital injection of tyloxapol and monitoring the appearance of triglyceride in the plasma over time. The rate of VLDL production was calculated from the slopes of the lines. All values are mean ± standard deviation. All mice used in these experiments were female.

*Dennd5b*^−/−^ mice and age-matched wildtype controls were placed on western diet (WD, 42% calories from fat and 0.2% cholesterol), beginning at 2 months of age. The rate of body weight gain in *Dennd5b*^−/−^ mice was about half the rate of WT mice (Fig. 3A). After 4 months on WD, the body weight of *Dennd5b*^−/−^ mice was 30% lower than WT mice. The difference in body weight was explained by a shift in body mass composition, with *Dennd5b*^−/−^ mice having lower fat mass and greater lean mass (Fig. 3B). On standard chow diet, WT and knockout mice maintain similar body weight (Supplementary Fig. 5). On WD, fecal lipids were measured and although total fecal mass was increased in *Dennd5b*^−/−^ mice (Fig. 3C and Supplementary Fig. 6A), significant increases in total lipid content were not detected, although there was a trend toward increased total lipids in *Dennd5b*^−/−^ feces (Supplementary Fig. 6B). Measurement of specific lipid components revealed that fecal total cholesterol and triglyceride content were not different but fecal free fatty acid content was significantly increased in the knockout mice (Supplementary Fig. 6C–E). In plasma, TC and PL increased compared to baseline values in WT mice and there was no change in triglyceride. However, *Dennd5b*^−/−^ mice were resistant to WD-induced plasma lipid increases (Fig. 3D–F). While on WD, WT mice maintained a higher amount of HDL and accumulated a larger-sized population of cholesterol-rich lipoprotein, likely small LDL, that was absent in *Dennd5b*^−/−^ mice (Fig. 3G,H). The favorable plasma lipid and body composition profile in *Dennd5b*^−/−^ mice suggested a possible protective effect against diet-induced atherosclerosis. Analyses of atherosclerotic lesion area in mice after 4 months on WD showed significantly lower plaque burden in *Dennd5b*^−/−^ mice compared to wildtype controls (Fig. 3I).

### *Dennd5b*^−/−^ mice compensate for lack of dietary lipid by increasing hepatic VLDL production

To evaluate the effect of *Dennd5b* knockout on hepatic lipid metabolism in mice on WD, we extracted lipid from liver tissue. TC and FFA were significantly reduced in *Dennd5b*^−/−^ mouse liver and there was a trend toward decreased TG (Fig. 3J–L). The VLDL production by the liver was evaluated by measuring TG appearance in plasma of fasting mice after intravenous tyloxapol injection. *Dennd5b*^−/−^ mice had a 21% increase in rate of VLDL associated TG accumulation compared to WT (slope = 7.5 vs 6.18, p = 0.0098) (Fig. 3M).

### A common *DENND5B* variant is associated with body mass index in humans

**Figure 4:**
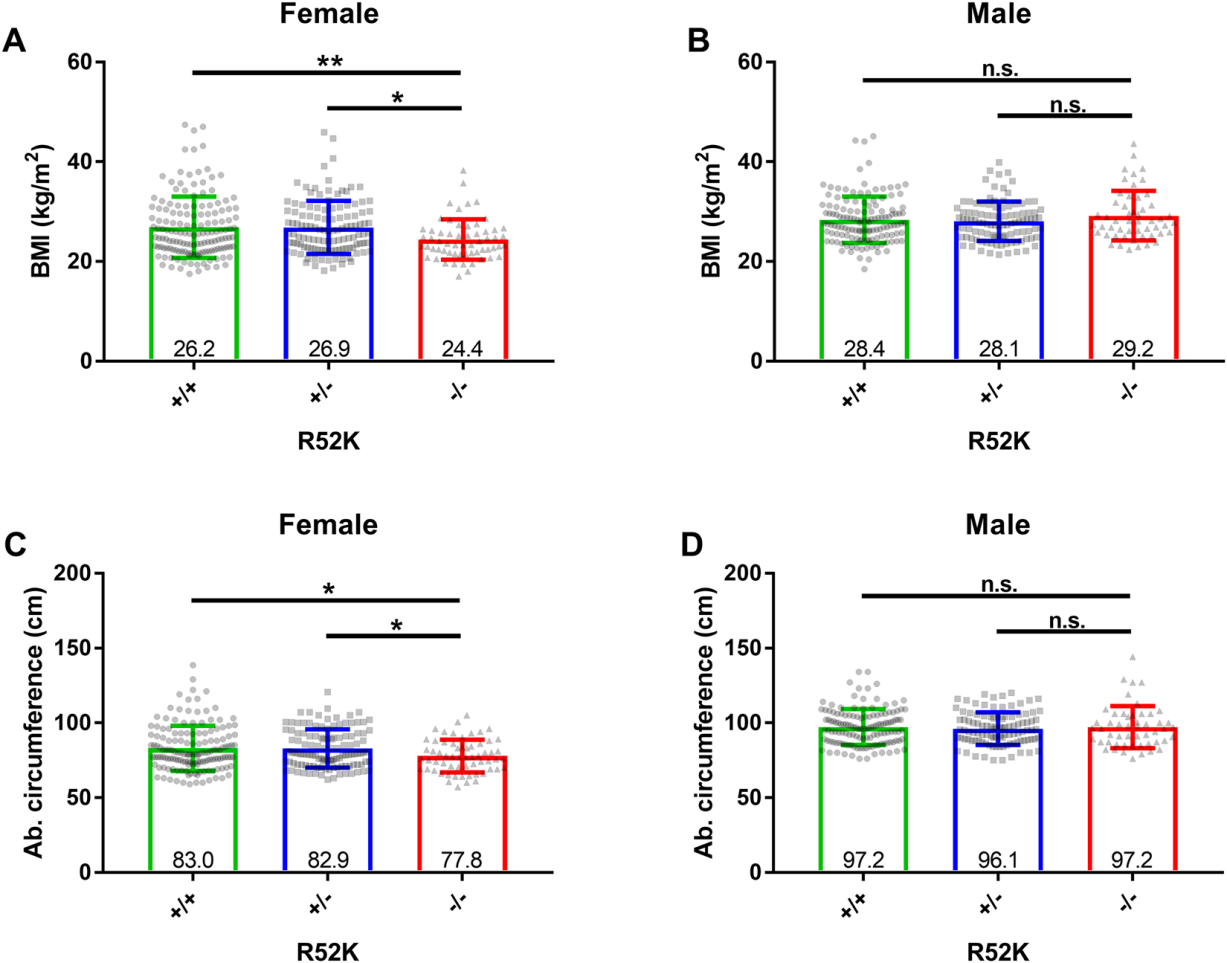
A common *DENND5B* genetic variant is associated with body mass index and abdominal circumference in humans. (**A,B**) An evaluation of body mass index (BMI) among wildtype, heterozygous, and homozygous carriers of the p.(R52K) rs4930979 variant in females (**A**) and males (**B**). (**C,D**) An evaluation of abdominal circumference among wildtype, heterozygous, and homozygous variant genotypes for the p.(R52K) variant in females (**C**) and males (**D**). Numbers at the base of each bar are the group mean. Statistical comparisons were performed by one-way ANOVA with post hoc correction by the Tukey method. *p < 0.05, **p < 0.01, n.s. = not significantly different. All values are mean ± standard deviation.

The human homologue (DENND5B) is 95% identical to the mouse protein. To determine if DENND5B plays a similar role in human physiology, we examined the influence of a common gene variant, p.(R52K) (rs4930979), on body weight and plasma lipids in the ClinSeq^®^ exome sequencing study (n = 621). Females homozygous for the variant allele had significantly lower BMI (24.4 vs. 26.9, p = 0.008, n = 329) and abdominal circumference (77.8 vs. 83.0, p = 0.03, n = 330), when compared to homozygous reference (Fig. 4A,C). The effect on BMI was due to increased body weight, no difference in height was observed. The p.(R52K) variant did not influence these measures in male participants (Fig. 4B,D) and did not correlate with plasma lipids for either gender in this cohort.

In an independent, larger cohort (Mayo Vascular Disease Biorepository, VDB), p.(R52K) (MAF = 0.3984) was associated with BMI (β = −0.179, *P* = 0.041, n = 8,303). In this dataset, another *DENND5B* variant p.(H487N) (rs1056320, MAF = 0.1072) was associated with LDL-C (β = 2.03, *P* = 0.0064, n = 8,571); however, this variant was not significantly associated with BMI (β = −0.217, *P* = 0.11, n = 8,303). One explanation for the differing metabolic presentations associated with these variants is that they have distinct influences on protein function due to their location in the protein. Perhaps DENND5B performs more than one function with influence on metabolism.

## Discussion

Overall, these data demonstrate a role for *Dennd5b* in murine enterocyte chylomicron secretion. In particular, *Dennd5b* appears to be critical in the Golgi to plasma membrane transport of chylomicron secretory vesicles. The metabolic consequence of genetic disruption of *Dennd5b* in mice is resistance to western diet induced increases in plasma lipids and body weight. Consistent with the observed phenotype in mice, we found that human variation in *DENND5B* is also associated with body weight and plasma lipids.

By electron microscopy, we were able to observe many of the key steps in the secretory pathway of chylomicrons. This allowed us to pinpoint the step of impairment in *Dennd5b*^−/−^ mice at the level of Golgi to plasma membrane transport of chylomicron secretory vesicles. The mechanisms of pre-Golgi and intra-Golgi processing of chylomicrons are well understood^6^; however, post-Golgi steps in the chylomicron secretory pathways are relatively uncharacterized. The *Dennd5b*^−/−^ mouse generated in this study, therefore, provides a unique and novel tool for further exploration of this important metabolic function.

Increased free fatty acid content in the feces of *Dennd5b*^−/−^ mice on WD supports the conclusion that impaired chylomicron secretion by the enterocyte results in an overall decreased absorption of dietary triglycerides. The appearance of electron dense, layered lipid structures by electron microscopy in *Dennd5b*^−/−^ mice after oil gavage suggests that the enterocyte does initially absorb fatty acid, but it accumulates intracellularly and shunts it to an autophagolytic pathway. This alternative pathway for triglyceride that is not secreted by enterocytes into the lymph may explain the lack of weight gain in the knockout mice on WD. In addition, unabsorbed free fatty acids in the intestine may likely be metabolized by gut bacteria, which may then have consequences on the gut microbiome and overall metabolism. Because chylomicrons are also important mediators of cholesterol absorption, the lack of increased fecal cholesterol excretion in *Dennd5b*^−/−^ mice was unexpected. Further studies will be required to elucidate the specific pathways involved in the metabolic disturbances observed in this model.

Increased hepatic VLDL production by *Dennd5b*^−/−^ mice may be a compensatory mechanism for peripheral triglyceride distribution under the circumstance of reduced availability of dietary lipids. This observation also prompts the conclusion that Dennd5b is not essential for the secretion of all apoB containing lipoproteins. This may suggest the existence of alternative post-Golgi mechanisms for hepatic VLDL secretion. Additionally, reduced lipid accumulation in *Dennd5b*^−/−^ liver could support a potential protective effect against non-alcoholic fatty liver disease.

Other members of the DENN domain-containing family of proteins may shed light on the details of DENND5B’s mechanism of action in chylomicron secretion. The DENN domains commonly contain GEF activity for Rab proteins, which are important effectors of intracellular vesicular trafficking^15^. Interestingly, one report that has characterized the GEF activities of the DENN has family of proteins found DENND5B to have GEF activity for Rab39^9^. Rab39 is also not well documented in the literature but has been reported as a Golgi-associated protein with involvement in intracellular trafficking^16^. As is true of all secretory processes, it is likely that there are many potential mediators involved in this pathway of Golgi to plasma membrane transport and fusion of chylomicron secretory vesicles. Future studies on the GEF activity of DENND5B may reveal important insight into the regulation of chylomicron secretory processes.

It is noteworthy that the reported mouse phenotype was only detected in homozygous knockouts. This suggests that *Dennd5b*-related dyslipidemia is inherited in an autosomal recessive pattern. In ClinSeq^®^, only subjects homozygous for the p.(R52K) minor allele had reduced BMI and abdominal circumference. Several *in silico* tools predict that this variant is benign (Mutation taster 0.0002 probability of pathogenicity, Polyphen2 0.0002 probability of pathogenicity, SIFT score 0.267 (tolerated)). That we observed a significant influence of this variant on relevant traits suggests either that the p.(R52K) variant itself has deleterious effects on DENND5B function in spite of the *in silico* predictors or that this variant is in linkage disequilibrium with another pathogenic variant in *DENND5B*.

In our mice, we confirmed GeneAtlas expression data indicating that *Dennd5b* is highly expressed in liver and intestine (Supplemental Fig. 1A). In the small intestine, expression levels were highest in duodenum and decreased in distal small intestine (Supplemental Fig. 1B). We observed a gender difference in hepatic *Dennd5b* expression. Female mice have significantly higher expression of Dennd5b in the liver, this may be related to the greater impact of *Dennd5b* deletion on circulating plasma lipid levels in female mice. Gender differences also appear to modulate the phenotype of this gene in humans. The effect of the p.(R52K) variant was only observed in females. Interestingly, the ExAC database^17^ shows only two heterozygous loss of function variants amongst ~60,000 individuals, which yields a pLI (Probability of Loss of Function Intolerance) score of 1.00 (the highest possible score). These data suggest that homozygous loss of function variants in the human cause a highly deleterious phenotype, which we hypothesize to be an essential function related to intracellular transport.

By influencing plasma lipids and body weight, DENND5B might be expected to affect cardiovascular disease risk, specifically atherosclerosis. In the knockout mouse, there was a reduction of total atherosclerosis burden. Although we did observe decrease atherosclerosis in the *Dennd5b*^−/−^ mouse compared to WT mice, the extent of atherosclerosis in WT mice was quite small. Future studies on *Dennd5b*^−/−^ crossed with *ApoE*^−/−^ or *Ldlr*^−/−^ are needed to better examine the role of *Dennd5b* in atherogenesis. There is also evidence of a possible association of *DENND5B* variants and atherosclerosis in humans. Levy *et al*. have found that miR-150-5p, which has a potential causal association with coronary heart disease, may act by regulating expression of *DENND5B,* among other target genes^18^. Furthermore, *in vitro* studies using the Huh7 human liver cell line have shown that another miRNA, miR-223, known to be associated with inflammation and cholesterol metabolism, causes significant downregulation of *DENND5B*^19^. Another study has reported an association between expression of *DENND5B* in whole blood and coronary artery disease in humans^20^. These studies suggest a complex regulatory network for *DENND5B* expression in humans and further highlight the potential importance of this gene in cardiovascular disease.

In summary, *DENND5B* was identified to play an important role in dietary lipid absorption (i.e., Golgi to plasma membrane trafficking of chylomicron secretory vesicles) and was found to also effect body weight, plasma lipid metabolism, and atherosclerotic cardiovascular disease in knockout mice. Further investigation into the mechanistic details of this activity may provide additional targets and alternative approaches to the treatment of metabolic and cardiovascular disease in humans.

## Supplementary information

Dataset 1

Dataset 2
