# Supplementary material for: DENND5B Regulates Intestinal Triglyceride Absorption and Body Mass
Source: Sci Rep. 2019 Mar 5;9:3597. doi: 10.1038/s41598-019-40296-0 (PMC6401118; doi:10.1038/s41598-019-40296-0)
Supplement: Supplementary file 1 — Dataset 1 [file 41598_2019_40296_MOESM1_ESM.docx]

***DENND5B* Regulates Intestinal Triglyceride Absorption and Body Mass**

Scott M. Gordon ^1,2*^, Edward B. Neufeld ^1^, Zhihong Yang ^1^, Milton Pryor ^1^, Lita A. Freeman ^1^, Xiao Fan ^3^, Iftikhar J. Kullo ^3^, Leslie G. Biesecker ^4^, Alan T. Remaley ^1^

^1^Translational Vascular Medicine Branch, National Heart, Lung, and Blood Institute, NIH, Bethesda, Maryland, 20892; USA.

^2^Saha Cardiovascular Research Center and Department of Physiology, University of Kentucky College of Medicine, Lexington, KY, 40536; USA.

^3^Department of Cardiovascular Diseases, Mayo Clinic, Rochester, Minnesota, 55905; USA

^4^Medical Genomics and Metabolic Genetics Branch, National Human Genome Research Institute, NIH, Bethesda, Maryland, 20892; USA

*Corresponding author: Scott M. Gordon ([scott.gordon@uky.edu](mailto:scott.gordon@uky.edu))

**Supplementary Figure 1**

**A**

**B**

**Expression of Dennd5b in wildtype mice.** (A) *Dennd5b* tissue expression data extracted from GeneAtlas ^1^ dataset MOE430, gcrma. (B) Tissues were harvested from wildtype mice on chow diet and stored in RNALater (Invitrogen) until RNA purification. Relative gene expression was quantified using TaqMan gene expression assays (ThermoFisher) for *Dennd5b* (assay ID: Mm00623234_m1) and normalized to *Actb* expression (assay ID: Mm02619580_g1) on an Applied Biosystems 7900HT instrument.

*** p<0.001 by 2-way ANOVA

**Supplementary Figure 2**

**(High-resolution version provided in Supplementary Dataset 2)**

**
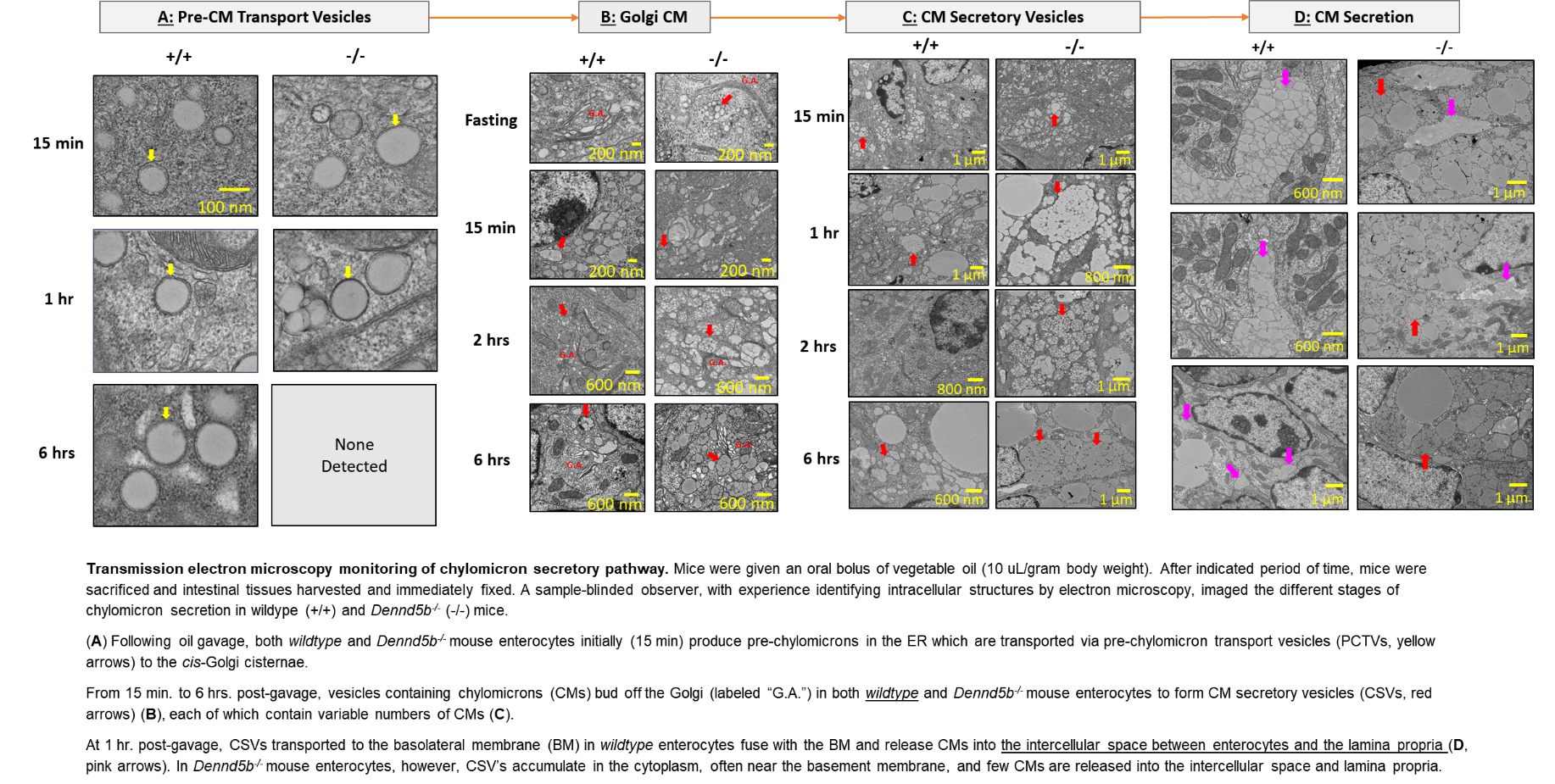
**

**Supplementary Figure 3**

**Transmission electron microscopy of enterocytes following oral oil gavage.** Mice were given an oral bolus of vegetable oil (10 uL/gram body weight). After the indicated periods of time, mice were sacrificed and intestinal tissues were harvested and immediately fixed and processed for TEM. At 15 min. post-gavage, pre-chylomicron transport vesicles (PCTVs, *yellow* arrows) in the apical cytoplasm as well as well as some lipid droplets (LDs, *green* arrows) were observed in both *wildtype* and *Dennd5b^-/-^* mouse enterocytes. After 1 hr., PCTVs, chylomicrons (CMs) in the Golgi cisternae, budding CSVs from *trans*-Golgi, and paranuclear CSVs were observed in both *wildtype* and *Dennd5b^-/-^* mouse enterocytes. In *wildtype* mouse enterocytes, modest LD formation was observed together with robust secretion of CMs into the intercellular space and lamina propria. In marked contrast, in *Dennd5b^-/-^* mouse enterocytes, massive accumulation of both LDs and CSVs were observed, with little or no secretion of CSVs into either the intercellular space or lamina propria. By 6 hrs. post-gavage, *wildtype* mouse enterocytes still contained some PCTVs, budding of CSVs from *trans*-Golgi, and some paranuclear CSVs. Few LDs remained in the cytoplasm. In marked contrast, in *Dennd5b^-/-^* mouse enterocytes, no PCTVs were observed, along with little if any Golgi CM formation, and massive LD and CSV accumulation. Most CMs in CSVs appeared to lose their structural integrity. Under fasting conditions, evidence of autophagy (blue arrow) was often present.

**15 min**

**Fasting**

***Dennd5b^-/-^***


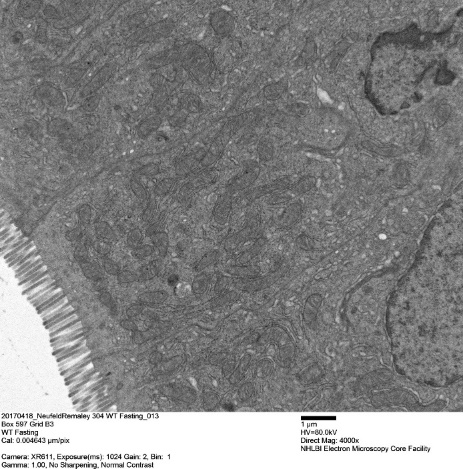

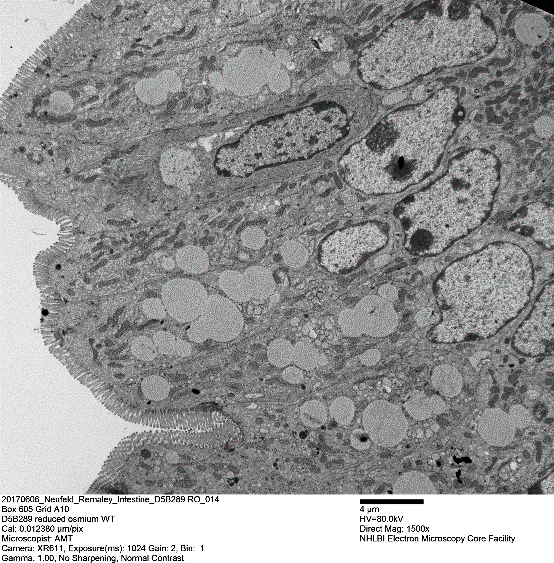

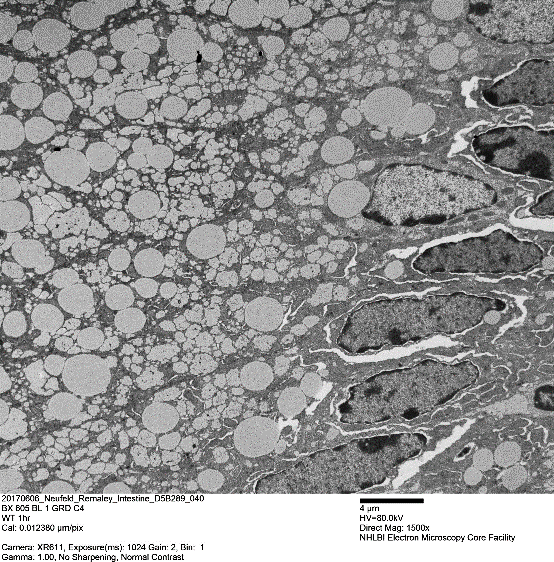

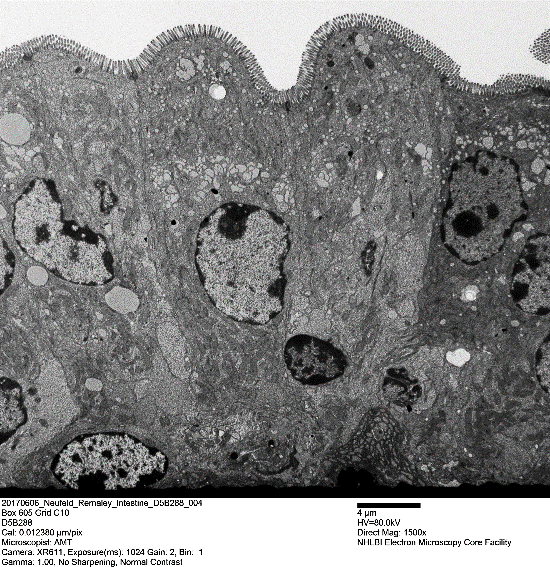

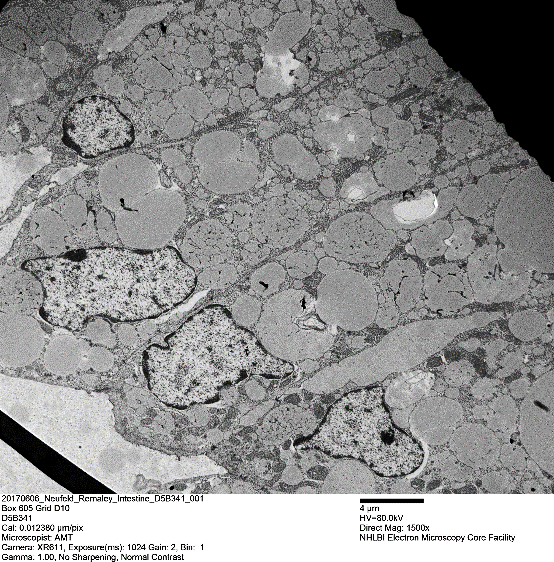

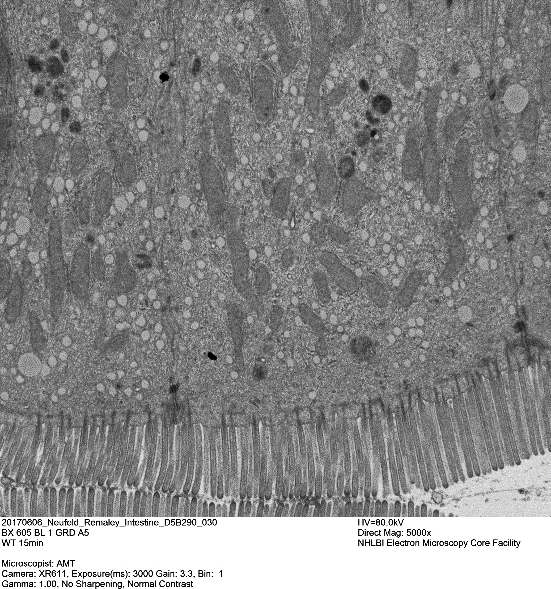

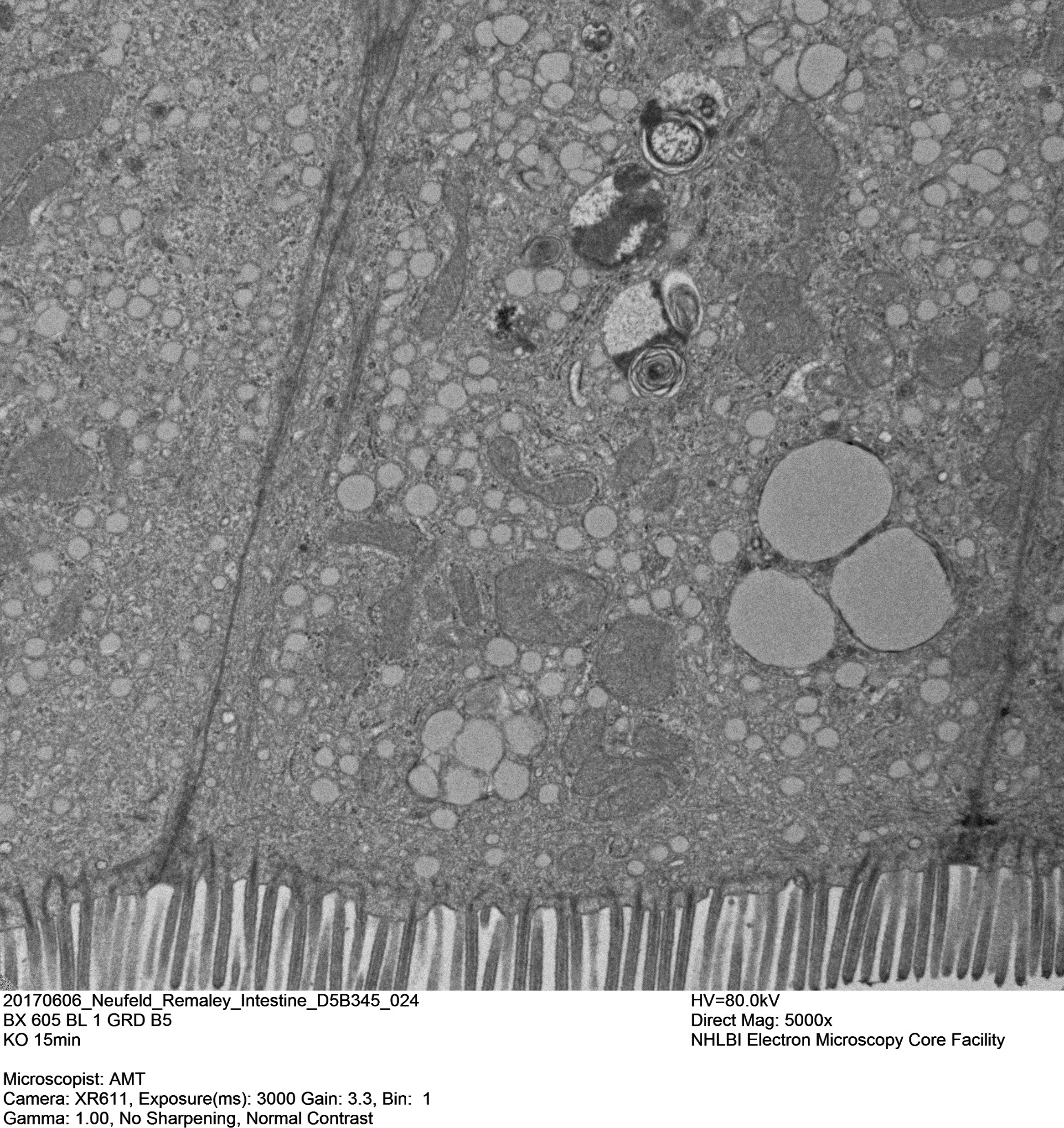


**1 hr**

**6 hrs**


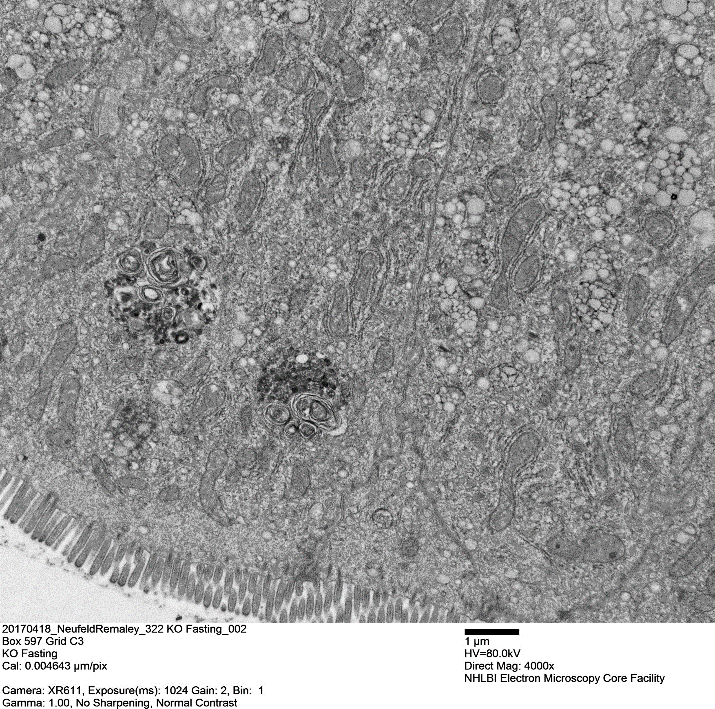


***Dennd5b^+/+^***

1 µm

1 µm

4 µm

4 µm

1 µm

1 µm

4 µm

4 µm

**Supplementary Figure 4**

**High magnification transmission electron micrograph of autophagosome in fasting *Dennd5b^-/-^* enterocyte.** These structures were frequently detected in *Dennd5b^-/-^* but not in wildtype enterocytes.

**Supplementary Figure 5**

**Body weight of wildtype and *Dennd5b^-/-^* mice on chow or western diet.** Wildtype (green) or *Dennd5b^-/-^* (red) mice were kept on either chow or western diet for 4 months (beginning at 2 months of age) and body weight measured. n = 6-8/group. Statistical analyses were performed using 2-way ANOVA with Sidak post hoc test. **** p < 0.0001; n.s. = not significantly different. All values are mean ± standard deviation.

**Supplementary Figure 6**


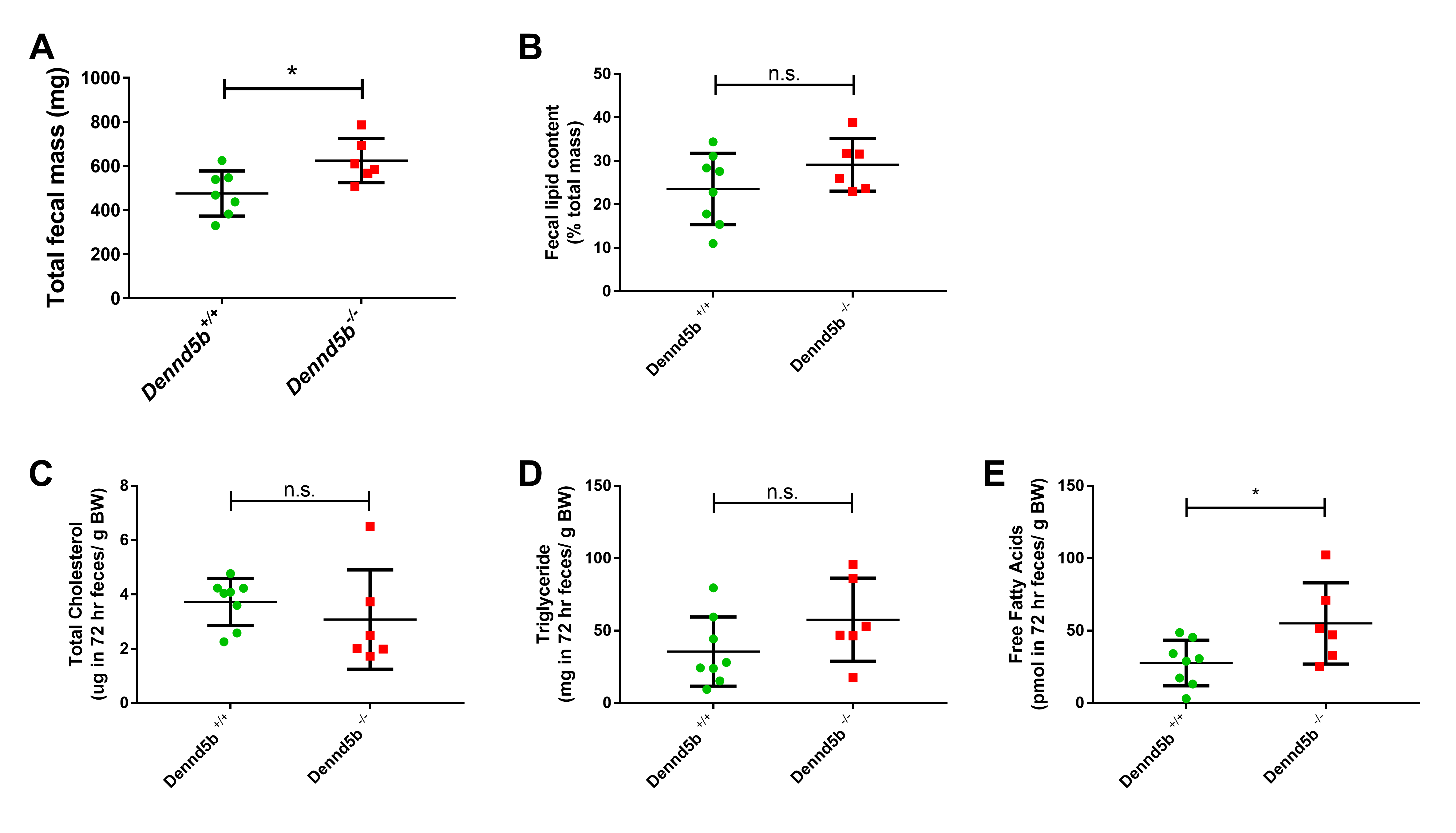


**Fecal lipid mass and composition in wildtype and *Dennd5b*^-/-^ mice on WD.** Wildtype (green) or *Dennd5b*^-/-^ (red) mice were kept on WD for 4 months (beginning at 2 months of age). Feces were collected over 72 hours, dried, and total mass was measured (A). Feces were also analyzed for total lipid content (B). Lipids were extracted and total cholesterol, triglyceride, and free fatty acids were measured. These values are reported as the total mass collected over 72 hours adjusted for body weight (C-E). n = 6-8/group. Statistical analyses were performed using unpaired t tests. * p <0.05, n.s. = not significantly different. All values are mean ± standard deviation.

**Supplementary Figure 7**

**
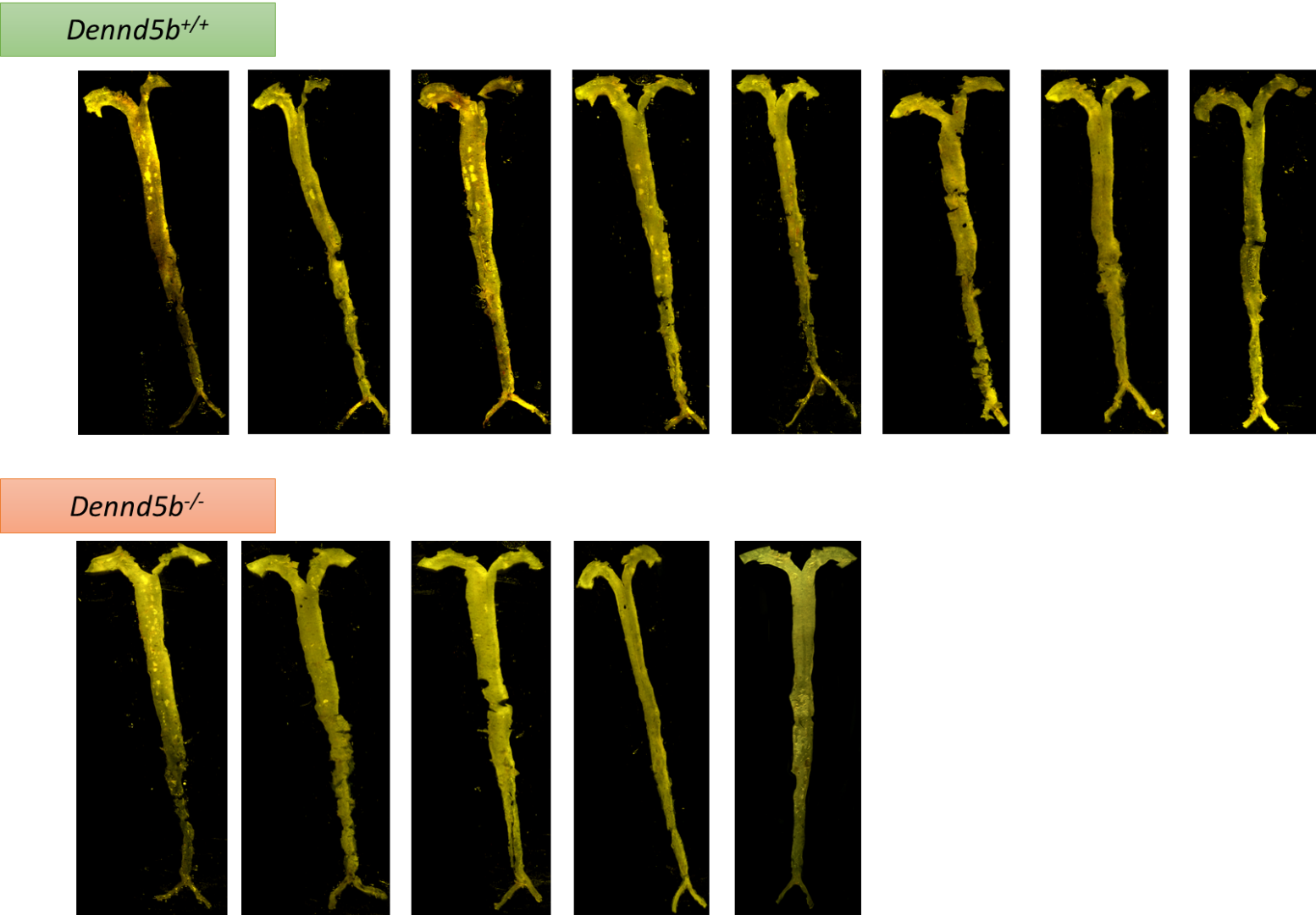
**

**Atherosclerosis lesion analysis in mice on WD.** Wildtype or *Dennd5b^-/-^* mice were maintained on WD for 4 months. Aortas were harvested and stained with Sudan IV to detect atherosclerotic lesion area.

**References**

1. Su, A.I., et al. A gene atlas of the mouse and human protein-encoding transcriptomes. Proceedings of the National Academy of Sciences of the United States of America 101, 6062-6067 (2004).
