# Supplementary material for: DENND5B Regulates Intestinal Triglyceride Absorption and Body Mass
Source: Sci Rep. 2019 Mar 5;9:3597. doi: 10.1038/s41598-019-40296-0 (PMC6401118; doi:10.1038/s41598-019-40296-0)
Supplement: Supplementary file 2 — Dataset 2 [file 41598_2019_40296_MOESM2_ESM.docx]

***DENND5B* Regulates Intestinal Triglyceride Absorption and Body Mass**

Scott M. Gordon ^1,2*^, Edward B. Neufeld ^1^, Zhihong Yang ^1^, Milton Pryor ^1^, Lita A. Freeman ^1^, Xiao Fan ^3^, Iftikhar J. Kullo ^3^, Leslie G. Biesecker ^4^, Alan T. Remaley ^1^

^1^Translational Vascular Medicine Branch, National Heart, Lung, and Blood Institute, NIH, Bethesda, Maryland, 20892; USA.

^2^Saha Cardiovascular Research Center and Department of Physiology, University of Kentucky College of Medicine, Lexington, KY, 40536; USA.

^3^Department of Cardiovascular Diseases, Mayo Clinic, Rochester, Minnesota, 55905; USA

^4^Medical Genomics and Metabolic Genetics Branch, National Human Genome Research Institute, NIH, Bethesda, Maryland, 20892; USA

*Corresponding author: Scott M. Gordon ([scott.gordon@uky.edu](mailto:scott.gordon@uky.edu))

**Transmission electron microscopy monitoring of chylomicron secretory pathway.** Mice were given an oral bolus of vegetable oil (10 uL/gram body weight). After indicated period of time, mice were sacrificed and intestinal tissues harvested and immediately fixed. A sample-blinded observer, with experience identifying intracellular structures by electron microscopy, imaged the different stages of chylomicron secretion in wildype (+/+) and *Dennd5b^-/-^* (-/-) mice.

(**A**) Following oil gavage, both *wildtype* and *Dennd5b^-/-^* mouse enterocytes initially (15 min) produce pre-chylomicrons in the ER which are transported via pre-chylomicron transport vesicles (PCTVs, yellow arrows) to the *cis*-Golgi cisternae.

From 15 min. to 6 hrs. post-gavage, vesicles containing chylomicrons (CMs) bud off the Golgi (labeled “G.A.”) in both *wildtype* and *Dennd5b^-/-^* mouse enterocytes to form CM secretory vesicles (CSVs, red arrows) (**B**), each of which contain variable numbers of CMs (**C**).

At 1 hr. post-gavage, CSVs transported to the basolateral membrane (BM) in *wildtype* enterocytes fuse with the BM and release CMs into the intercellular space between enterocytes and the lamina propria (**D**, pink arrows). In *Dennd5b^-/-^* mouse enterocytes, however, CSV’s accumulate in the cytoplasm, often near the basement membrane, and few CMs are released into the intercellular space and lamina propria.

**A: Pre-CM Transport Vesicles**

**15 min**


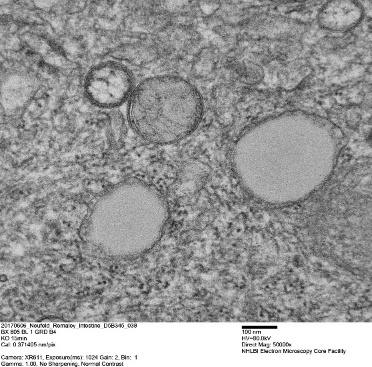

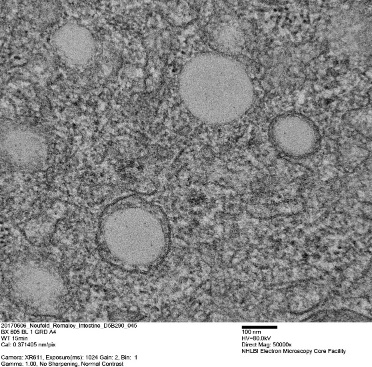


**+/+**


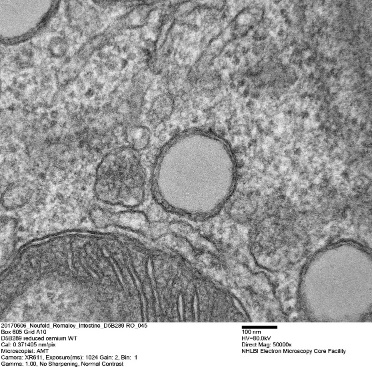

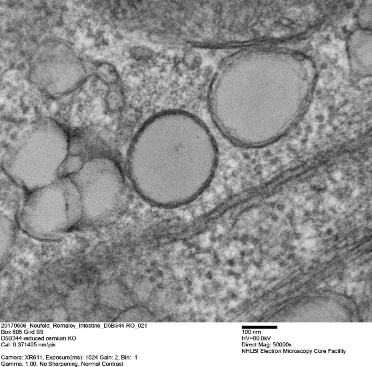

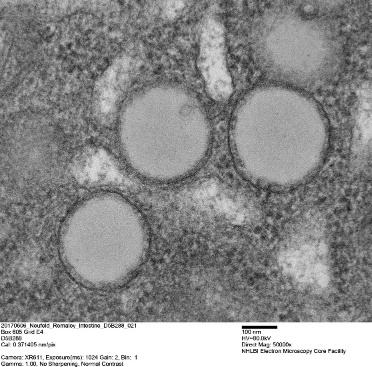


None

Detected

**1 hr**

**6 hrs**

**-/-**

100 nm

**B: Golgi CM**

**C: CM Secretory Vesicles**


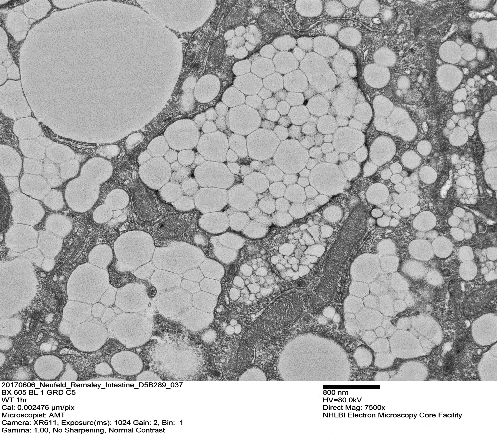

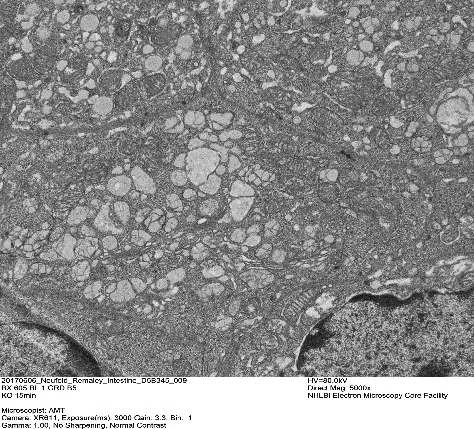

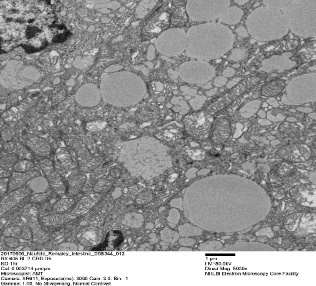


**15 min**

**1 hr**

**2 hrs**


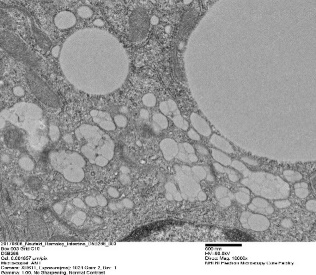

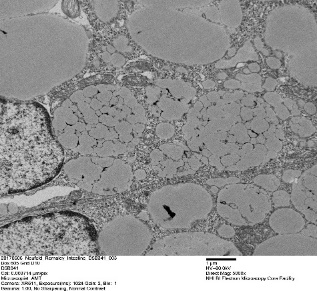


**6 hrs**

**+/+**

**-/-**


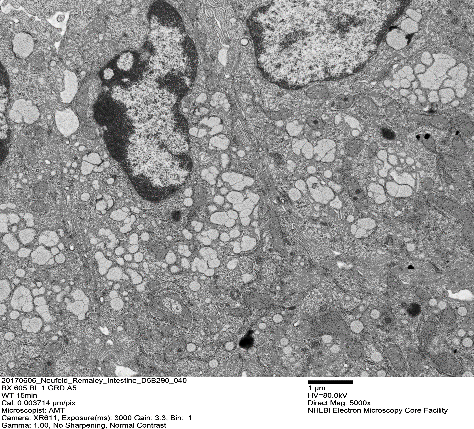


1 µm

1 µm

1 µm

800 nm


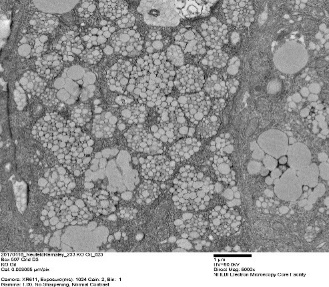


1 µm


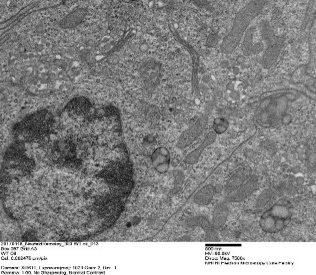


800 nm

600 nm

1 µm


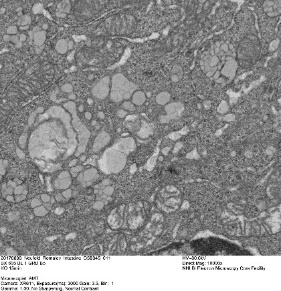

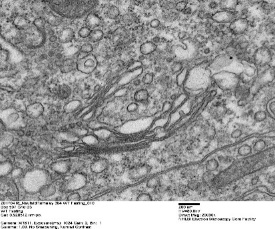

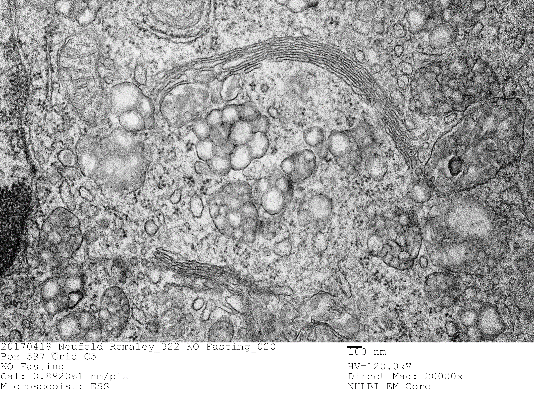


**+/+**

**-/-**

**15 min**

**Fasting**

**2 hrs**

**6 hrs**


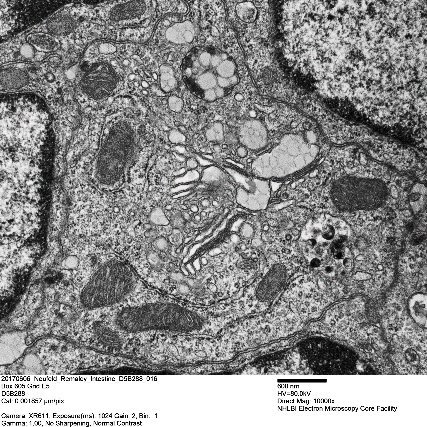

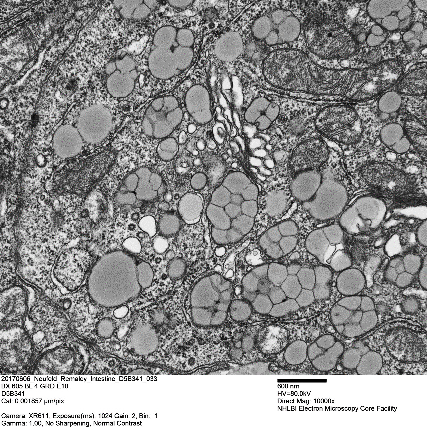

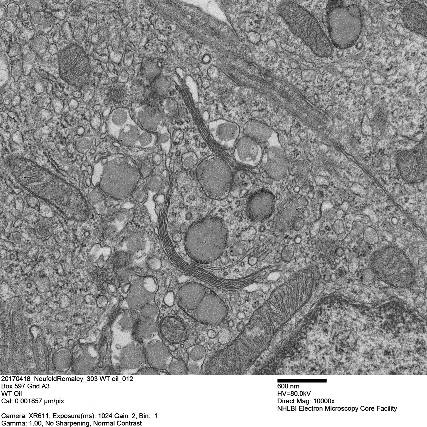

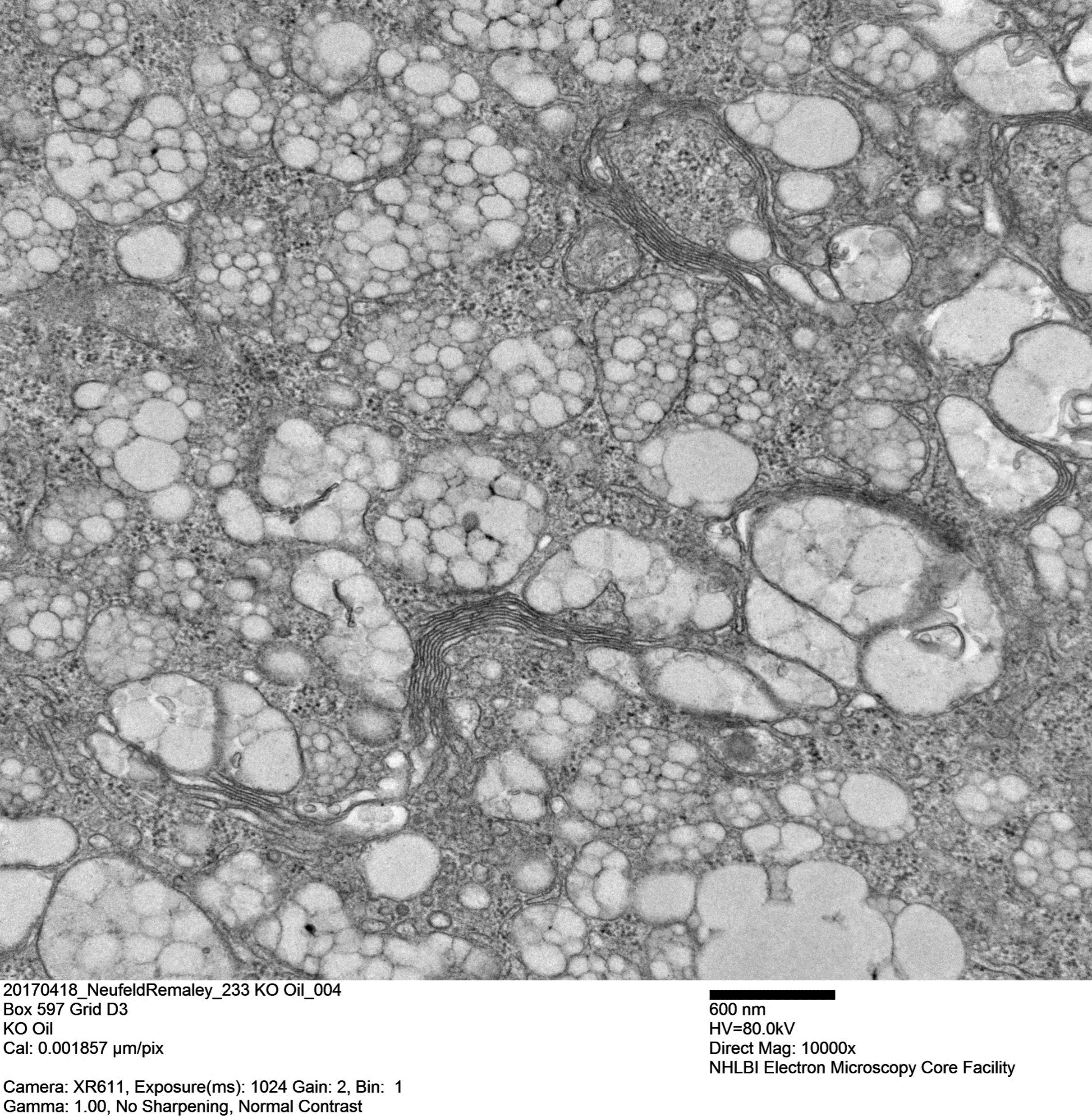

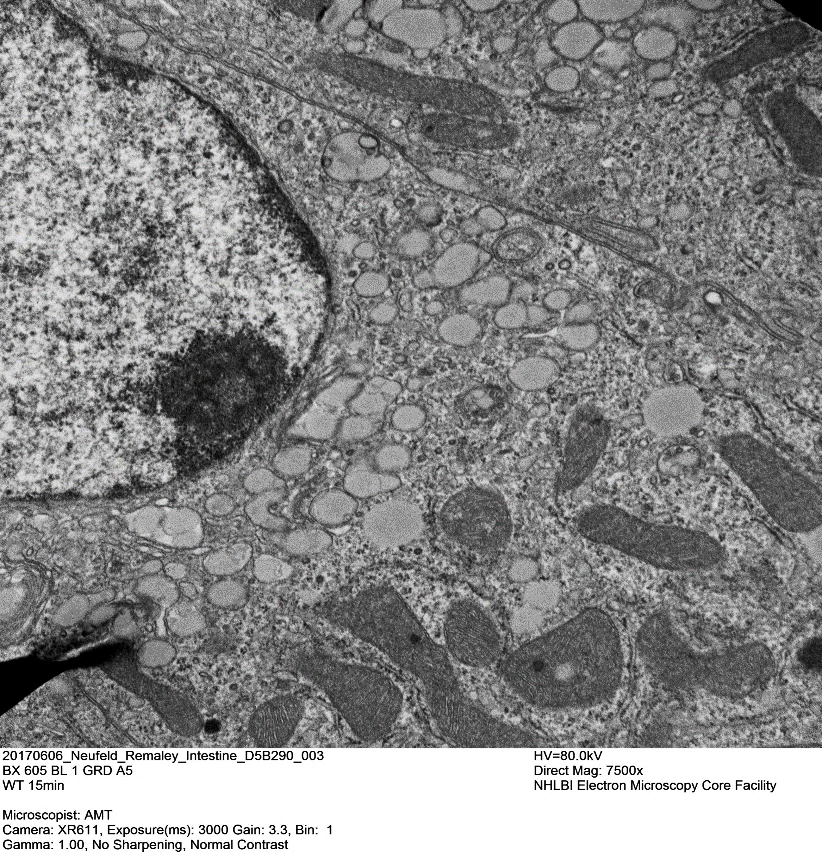


200 nm

200 nm

600 nm

600 nm

600 nm

600 nm

**D: CM Secretion**

**-/-**

**+/+**

200 nm

200 nm

**Supplementary Figure 2**

**G.A.**

**G.A.**

**G.A.**

**G.A.**

**G.A.**

**G.A.**


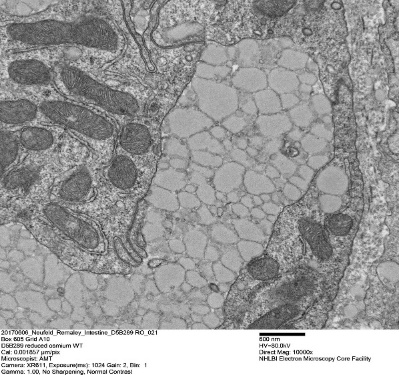

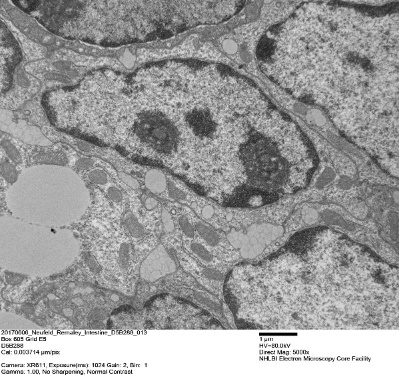

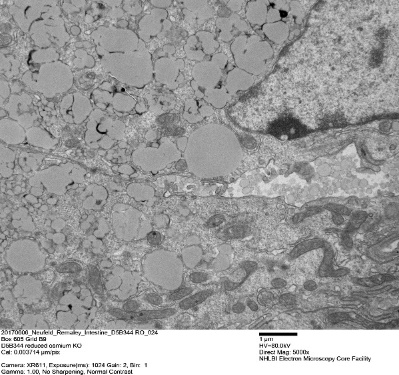

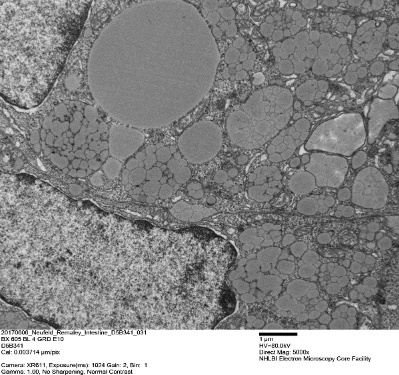

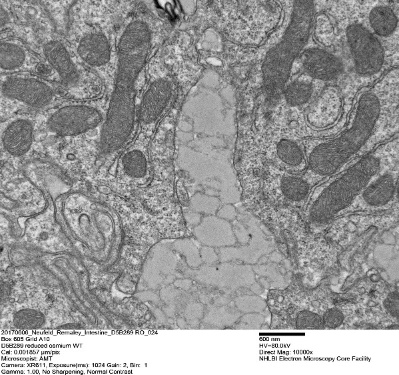

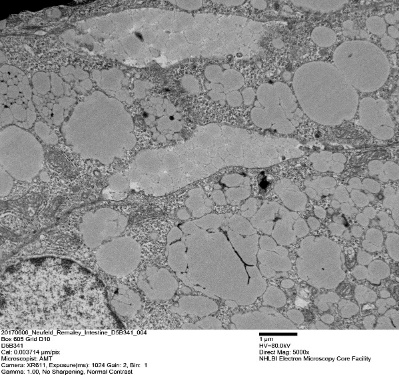


600 nm

1 µm

600 nm

1 µm

1 µm

1 µm
